# Clinical Oral Condition Analysis and the Influence of Highly Active Antiretroviral Therapy on Human Salivary Microbial Community Diversity in HIV-Infected/AIDS Patients

**DOI:** 10.3389/fcimb.2022.937039

**Published:** 2022-06-29

**Authors:** Peilin Cao, Yifan Zhang, Guangyan Dong, Hongkun Wu, Yuxiang Yang, Yi Liu

**Affiliations:** ^1^ Department of Stomatology, Sichuan Provincial People’s Hospital, University of Electronic Science and Technology of China, Chengdu, China; ^2^ State Key Laboratory of Oral Diseases, National Clinical Research Center for Oral Diseases, West China Hospital of Stomatology, Sichuan University, Chengdu, China; ^3^ Department of Stomatology, Hangzhou Dental Hospital Group, Hangzhou, China; ^4^ Division of Advanced Prosthetic Dentistry, Tohoku University Graduate School of Dentistry, Sendai, Japan; ^5^ Department of Radiology, West China School of Public Health and West China Fourth Hospital, Sichuan University, Chengdu, China

**Keywords:** acquired immune deficiency syndrome, oral characterization, oral microbiota, roche 454 sequencing, highly active antiretroviral therapy

## Abstract

The purpose of this study was to assess the clinical oral status and investigate the effect of highly active antiretroviral therapy (HAART) on oral flora diversity in human immunodeficiency virus (HIV)-infected/acquired immune deficiency syndrome (AIDS) patients. We first recorded and analyzed the demographic indicators of 108 HIV-infected patients and assessed their periodontal health, dental health and oral lesion status by oral examination. Besides, we compared the changes in salivary microbial communities of healthy controls, before and after treatment of HAART-processed AIDS patients by Roche 454 sequencing and RT-qPCR. In HIV-infected/AIDS patients, age, sex, marital status, income level, smoking and oral health behaviors had an effect on periodontal clinical indicators; age and marital status were correlated with dental clinical indicators; most of them were accompanied by oral manifestations, mainly including candidiasis albicans, salivary gland disease, AIDS-associated periodontitis, and oral ulcers. Besides, a total of 487 species were detected in the saliva of AIDS patients. The microbial communities of HAART-unprocessed AIDS patients significantly differed from those processed patients, with 112 unique microbial species. More importantly, a large number of conditioned pathogens were also detected in the saliva samples of AIDS patients, which may be associated with opportunistic infections. Therefore, HAART might have a crucial role in salivary microecological balance in AIDS patients. And these patients should pay attention to the maintenance of oral health, and the early initiation of HAART may be important for the development of oral lesions.

## Introduction

Acquired immune deficiency syndrome (AIDS) is caused by infection with the human immunodeficiency virus (HIV) (Upreti et al., 2020; Chen and Wang, 2022). HIV can attack and disrupt the immune system microbalance, producing a range of signs and symptoms (Bbosa et al., 2019). Patients with AIDS are highly susceptible to various bacterial or viral infections due to their severely compromised immune function (Meer, 2019). Besides, HIV infection can be combined with various opportunistic infections and rare tumors, leading to death (Cilliers et al., 2019; Chen and Wang, 2022). In recent years, the number of HIV infections worldwide is increasing and has become an increasingly serious social problem (Lapointe and Harrigan, 2020). It has been reported that 30% of HIV patients first present with oral symptoms, which will cause oral and maxillofacial infections after immune compromise (Hulgan and Samuels, 2021). Within 1-4 years prior to the onset of AIDS, patients may present with various typical oral lesions (Peacock et al., 2017). This has also become a key symptom for the early detection and diagnosis of AIDS. Currently, the international medical community attaches great importance to the oral manifestations of HIV. Therefore, it is of scientific importance to further investigate the oral representation of AIDS and its potential treatment strategies.

Highly active antiretroviral therapy (HAART) is the most effective therapy for HIV infection (Lu et al., 2018). And the effective implementation of HAART therapy can greatly reduce the opportunistic infections in AIDS patients, and decrease the morbidity and mortality of HIV-infected patients (Andrade et al., 2017). A decreasing trend has been reported in the incidence of HIV/AIDS-related oral diseases after the introduction of HAART (Oliva-Moreno and Trapero-Bertran, 2018). Research also testified that HIV/AIDS-related oral disease can be applied as one of the indicators to evaluate the efficacy of HAART (Shekatkar et al., 2021). Additionally, studies confirmed that the development of oral diseases is relevant to the oral microbial community (Graves et al., 2019; Mosaddad et al., 2019). The oral cavity is inhabited by vast commensal and pathogenic microorganisms (Gao et al., 2018). Dysbiosis of ecological balance is the initiating factor in the progression of oral infectious diseases (Zhang et al., 2018). The imbalance of local and systemic immunity and metabolism in HIV-infected/AIDS patients can cause an imbalance in oral ecology, which often result in multiple infectious diseases (Tappuni and Sufiawati, 2020). However, the alteration of the oral microbial community in AIDS patients after HAART is not completely clear. Therefore, it is crucial to explore the changes and characteristics of oral microorganisms in AIDS patients during HAART for the evaluation of HAART efficacy.

Currently, 454 pyrophosphate sequencing can enable deep sequencing of microorganisms in the oral environment, which also can identify the changes in the structure of the oral microbiome (Keijser et al., 2008; Yan et al., 2016). This has an essential role in exploring the dynamic relationship between oral microorganisms and changes in the oral environment and systemic health status. The Roche 454 GS FLX+ is the latest upgraded high-throughput sequencing system from 454 Life Science (Forgetta et al., 2013). And its maximum read length can reach 1000bp, and the sequencing accuracy reaches 99.997% (Allali et al., 2017). This technique has been successfully applied to study the microbiome of oral samples, such as saliva, plaque, pulp infections, etc (Keijser et al., 2008; Nasidze et al., 2009; Lazarevic et al., 2010; Li et al., 2010; Ling et al., 2010).

Above all, we collected the demographic characteristics of HIV-infected patients through questionnaires and conducted detailed oral examinations of these patients, thereby assessing the relationship between patient demographic risk factors and periodontal or dental health status. Besides, we also adopted the Roche 454 GS FLX+ sequencing platform to compare the differences in salivary microbial communities among normal controls, before and after treatment of HAART-processed AIDS patient. Furthermore, we investigate the oral health and oral microbalance in HIV-infected patients. Therefore, the study will be clinically important for the disease control of HIV-infected patients and the efficacy monitoring of HARRT treatment. This will also have key clinical implications for monitoring the efficacy of HARRT treatment in AIDS patients.

## Materials and Methods

### Patient Samples

108 HIV-infected patients were obtained from the Public Health Clinical Medical Center (Chengdu, Sichuan, China). The informed consent forms were signed by all patients. Inclusion criteria included being 18 years of age and older, having the ability to express themselves, and meeting the diagnostic criteria for HIV-infected patients and AIDS patients (Volberding et al., 2012). Exclusion criteria included co-infection with serious opportunistic infections; co-infection with other serious or unstable chronic diseases; inability to move autonomously; and women during pregnancy. All study subjects completed an epidemiological questionnaire before undergoing clinical examination. This study also received the approval of the ethics committee in West China Hospital of Stomatology, Sichuan University (WCHSIRB-D-2015-004).

### Clinical Assessment of Periodontal Health

All enrolled patients underwent a comprehensive periodontal examination using Hu-Friedy William Probe and disposable mouth mirror. Periodontal clinical examination was performed by 4 dentists based on the Carranza’s Clinical Periodontology method (Newman et al., 2011). And the examination indexes contain probing Depth (PD), clinical attachment loss (CAL), CAL > 3 mm, and CAL > 5 mm, PD > 4 mm, and bleeding on probing (BOP). For PD and CAL, six locis (Proximal mid-buccal loci, buccal loci, distal mid-buccal loci, distal mid lingual loci, lingual loci and proximal mid-lingual loci) of each tooth were evaluated using a periodontal probe (Williams probe; Hu-Friedy; Hu-Friedy, Chicago, IL, USA). For BOP: a total of 4 locis in distal mid-buccal, median, proximal mid and lingual locis were examined. 0 = no bleeding, 1 = bleeding.

### Clinical Assessment of Dental Health

The dental examination method was consistent with periodontal examination. In line with the World Health Organization (WHO) method of oral health survey (Newman et al., 2011), natural teeth were counted as 28 teeth, excluding upper and lower third molars, and missing teeth were recorded as missing. Non-carious teeth: complete teeth without fillings and without filling treatment. Caries: sulcus or smooth surface of the tooth with a softened lesion at the base, and potential damage to the enamel or softening of the sulcus wall (Liu et al., 2020). The dental examination included number of permanent sound teeth, number of missing teeth from oral disease, number of decayed, missing, filled teeth (DMFT), and Number of decayed, missing, filled surfaces (DMFS).

### Oral Mucosa Examination

Fungal culture was performed in oral mucosal scrapings from the included patients. The examination was uniformly conducted using the WHO-recommended CPI probe and planar mouth mirror. Oral mucosal disease is diagnosed by referring to the criteria developed by the WHO collaborating centre for oral manifestations of HIV infection (Pinna et al., 2019; Indrastiti et al., 2020).

### Experimental Grouping

The study population was randomly divided into healthy control group (H, n = 78), AIDS group (before treatment of HAART-processed AIDS patients (UT), n = 64), and HAART treatment group (after treatment of HAART-processed AIDS patients (T), n = 62). Healthy controls were age-sex matched healthy people, which were chosen at random. And inclusion criteria for healthy controls included 18 years of age or older, HIV antibody test was negative, having the ability to express themselves, informed consent, voluntary participation. Exclusion criteria included combination of fatal opportunistic infections, combination of various malignancies, combined with cranial infection, unconsciousness, inability to move on their own, women during pregnancy.

### Bacterial Total Genomic DNA Extraction

Referring to the method described in previous study, 5 mL of non-stimulated saliva samples were collected and stored in a -80°C refrigerator (Wong, 2009). Based on the kit instruction and previous research (Tian et al., 2010), the collected saliva samples were subjected to extraction of total bacterial genomic DNAs through the MasterPure™ DNA Purification Kit (Epicentre, Madison, USA) after optimizing the conditions. Briefly, an equal volume of saliva was mixed with PBS, centrifuged, and the supernatant was collected. The supernatant was centrifuged at 14,000 × g for 10 min at 4°C and the precipitate was retained. The precipitate was added to 299 µL of cell lysate and 1 µL of 50 µg proteinase K in a 65°C water bath for 40 min. The sample was cooled to 37°C, and added with 1.5 µL of RNase A in a 37°C water bath for 30 min. After 3 min on ice, 175 µL of MPC Protein Precipitation Reagent was added to the sample and centrifuged at 14,000 × g for 10 min at 4°C. the precipitate was added to 500 µL of isopropanol and centrifuged. The precipitate was washed with 75% alcohol and the DNA was lysed with 20 μL TE buffer.

### Roche 454 Sequencing

Each group contained 5 samples were collected for Roche 454 sequencing. The bacterial genomic DNA was first amplified using multiple pairs primers. And we selected the universal primer 347F/803R for the V3-V5 region of the bacterial 16S rDNA gene (Nossa et al., 2010). Roche 454 universal junction primers (454 A and B), multiplex identifier (MID) and target amplification primers were integrated as fusion primers. Then genomic DNA was amplified by multiplex PCR through the FastStart High Fidelity PCR System (Roche Applied Science, Mannheim, Germany). The amplification product is the amplicon library. Next, the libraries were purified using Agencourt AMPure XP PCR (Agencourt Bioscience, MA, USA). The libraries were assayed for fragmentation and concentration using LabChip GX (Caliper Life Sciences, MA, USA). Libraries were emulsified with GS FLX Titanium emPCR Kit (454 Life Sciences, CT, USA). And the library was sent to the GS FLX Titanium sequencing platform (454 Life Sciences, CT, USA) for pyrosequencing.

### Quantitative Real-Time PCR Analysis

Quantitative real-time PCR (RT-qPCR) assays were performed using the 16S rRNA gene as the target and using PCR amplification primers and hydrolysis-probe detection on the Light Cycler 96 (Roche, Basel, Switzerland). Each microbial DNA RT-qPCR array plate analyzed one sample for 93 species (NCBI Tax ID)/gene at a time. Overall bacterial load and host genomic DNA were measured as internal reference (Korona-Glowniak et al., 2021).

### Composition and Abundance Analysis of Operation Taxonomy Unit in Microbial Community

The sequences of 16S rDNA V3-V5 region were sequenced to obtain the raw sequence data, which were then processed using Mothur (Version 1.32.1, http://www.mothur.org/) to acquire Unique Reads. And then the operation taxonomy unit (OUT) with species were annotated (Schloss et al., 2009). The VennDiagram package in R language was applied to produce the Venn diagram (Chen and Boutros, 2011). The heat map was produced using R language (v2.15.3, http://www.r-project.org/index.html). Phylogenetic trees were constructed and plotted using QIIME (Version 1.50, http://qiime.org/index.html) and R language.

### Diversity Analysis

α-diversity analysis refers to the analysis of species diversity in individual samples (Kemp and Aller, 2004). The α-diversity of samples was calculated using mothur (Schloss et al., 2009). Differences in α-diversity indices between groups were compared using R language and the corresponding dilution curves were plotted. The β-diversity can reflect the differences in species diversity of different samples, which was analyzed with QIIME (Crawford et al., 2009).

### Cluster Analysis of Species Composition

Using QIIME software, 75% of the reads were randomly chosen for calculation in each sample, and the final clustering tree was obtained after 100 iterations of comprehensive statistics, which was plotted using R language. Significant differences in microbial composition between groups were analyzed using Metastats software, and p-values were corrected for the Benjamini-Hochberg method using the R language (Crawford et al., 2009).

### Statistical Analysis

The clinical data was compared using non-parametric Kruskal Wallis test with SPSS 23.0 software (Inc., Chicago, IL). For α-diversity analysis, the Wilcoxon Rank-Sun test was applied for the comparison of two samples; the Kruskal-Wallis test was applied for the comparison of multiple samples. *P* < 0.05 signified that the difference was significant.

## Results

### Relationship Between Demographic Variables and Periodontal Clinical Indicators in HIV-Infected Patients

By analyzing the relationship between demographic variables and clinical indicators of periodontal health, we found that age was associated with probing depth, CAL, PD > 4 mm, CAL > 3 mm, and CAL > 5 mm in the study population (*P* < 0.01). Sex was relevant to probing depth and CAL only (*P* < 0.05), with the female group being higher than the male group. There was no statistically significant difference between ethnicity and periodontal clinical indicators. Marital status was related to probing depth, CAL, PD > 4 mm, and CAL > 5 mm (*P* < 0.05, *P* < 0.01), and all three indicators were lower in the unmarried group than in the other groups. Education and income per year were associated with probing depth, CAL, CAL > 3 mm and CAL > 5 mm (*P* < 0.05, *P* < 0.01). Residence had an effect on CAL only (*P* < 0.05) and was higher in the rural group than in the urban group. BMI was correlated with BOP-positive sites and probing depth (*P* < 0.05), and both indicators were also higher in the higher BMI group (**Table 1**). Besides, we found that alcohol consumption was relevant to CAL > 3 mm (*P* = 0.044), while the smoking population was statistically associated with probing depth, CAL, PD > 4 mm, CAL > 3 mm, and CAL > 5 mm. Moreover, we discovered that in the target population, frequency of tooth was only associated with BOP-positive sites (*P* = 0.035); Dental flossing was statistically associated with BOP-positive sites and CAL > 5 mm (*P* < 0.05). Dental visit experience was negatively associated with BOP-positive sites, PD, PD > 4 mm, CAL > 3 mm, and CAL > 5 mm (*P* < 0.05) (**Table 2**).

**Table 1:** Comparison of demographic variables with periodontal health status in HIV-infected patients, n = 108.

Variables	N	BOP (%)	Mean Probing Depth (mm)	Mean CAL (mm)	PD > 4 mm (%)	CAL>3mm (%)	CAL > 5 mm (%)
Age (years)	108						
13~24	26	36.47	1.58	0.59	3.40	7.02	2.66
25~44	32	33.29	2.75	2.46	14.43	31.40	9.94
45~64	29	34.74	4.28	4.11	24.62	41.74	13.49
≥65	21	29.84	3.88	3.88	17.36	39.06	12.38
	*p value*	0.275	0.000^**^	0.000^**^	0.000^**^	0.000^**^	0.000^**^
Sex	108						
Male	50	33.92	2.95	2.59	15.43	28.05	9.207
Female	58	32.73	3.75	3.36	13.71	37.33	11.41
	*p*	0.587	0.021^*^	0.046^*^	0.554	0.088	0.145
Ethnic	108						
Han	97	33.94	3.16	2.81	15.34	29.96	9.86
Minority	11	30.50	2.46	2.03	11.86	29.85	7.23
	*p value*	0.508	0.202	0.258	0.362	0.919	0.209
Marital status	108						
Single	19	31.94	2.23	1.50	8.42	19.16	3.95
Married	45	35.68	3.12	2.90	14.23	31.59	10.59
Divorced	25	33.58	3.68	3.47	18.63	35.02	11.61
Widowed	19	30.63	3.24	2.68	20.31	31.13	11.67
	*p value*	0.497	0.004^**^	0.001^**^	0.038^*^	0.088	0.001^**^
Education (years)	108						
<9	43	33.09	3.57	3.35	18.18	36.02	11.50
9~12	46	34.92	3.031	2.61	14.12	27.83	8.98
>12	19	31.41	2.03	1.43	9.16	19.04	6.47
	*p value*	0.463	0.000^**^	0.000^**^	0.077	0.000^**^	0.012^*^
Income per year (RMB)	108						
<12000	12	30.06	4.03	3.42	19.36	38.77	10.76
12000~36000	62	33.91	3.30	3.12	16.18	32.12	10.80
>36000	34	34.61	2.45	1.85	11.61	22.95	7.28
	*p value*	0.612	0.004^**^	0.001^**^	0.090	0.015^*^	0.038^*^
Residence	108						
Urban	38	33.14	2.79	2.27	14.41	25.40	8.46
Rural area	70	33.62	3.31	3.03	15.55	32.40	10.28
	*p value*	0.931	0.127	0.031^*^	0.560	0.070	0.183
BMI (kg/m^2^)	108						
<18.5	25	27.81	3.88	3.30	17.73	35.16	10.90
18.5~24	78	34.37	2.83	2.51	14.71	28.08	9.29
24~28	5	52.75	3.95	3.79	7.56	34.40	9.35
	*p value*	0.024^*^	0.006^**^	0.072	0.196	0.254	0.762

Non-parametric Kruskal Wallis test. *, P < 0.05; **, P < 0.01. BOP, Bleeding on probing; CAL, Clinical attachment loss; PD, Probing depth. Bold values means p < 0.05.

**Table 2:** Effect of oral health care behaviors on periodontal clinical indicators in HIV-infected patients, n = 108.

Variables	N	BOP	Probing Depth	Mean CAL	PD > 4 mm Sites	CAL > 3 mm Sites	CAL > 5 mm Sites
		(%)	(Mean, mm)	(mm)	(%)	(%)	(%)
Frequency of tooth brushing	108						
Twice a day	23	26.60	2.87	2.42	15.70	24.79	8.21
Once a day	76	35.07	3.11	2.80	15.00	30.88	9.89
Seldom/Never	9	45.05	4.43	3.55	13.29	39.63	12.88
	*P value*	0.035^*^	0.246	0.406	0.988	0.310	0.390
Dental flossing	108						
Once a day	14	27.47	2.58	2.17	10.10	22.54	5.62
Seldom/Never	94	34.78	3.21	2.85	15.96	31.26	10.38
	*p value*	0.034^*^	0.062	0.173	0.173	0.136	0.028^*^
Dental prophylaxis	108						
Once a year	4	11.5	2.10	1.77	5.82	22.90	3.97
Dentists’ advice	39	34.56	2.75	2.21	14.06	23.55	7.80
Never	65	34.33	3.39	3.12	16.20	34.21	11.09
	*p value*	0.025^*^	0.034^*^	0.058	0.207	0.026^*^	0.016^*^
Dental visit	108						
Less than a year	4	25.73	3.30	3.29	30.87	37.10	13.80
1~2 years ago	39	37.00	2.87	2.34	10.41	24.37	8.87
3~5 years ago	31	31.37	3.07	2.94	14.84	30.42	6.37
>5 years ago	34	32.98	3.36	2.95	18.47	34.48	10.89
	*p value*	0.503	0.408	0.529	0.042^*^	0.267	0.584

Non-parametric Kruskal Wallis test. *, P < 0.05. BOP, Bleeding on probing; CAL, Clinical attachment loss; PD, Probing depth. Bold values means p < 0.05.

### Oral Lesions in HIV-Infected Patients

Among the 108 HIV-infected patients, oral manifestations were higher in candidiasis albicans (47.23%), salivary gland disease (7.41%), AIDS-associated periodontitis (19.44%), and oral ulcers (20.37%), and low in oral hairy leukoplakia (1.85%) and herpes simplex stomatitis (3.70%). Lymphadenitis, Kaposi’s sarcoma, and non-Hodgkin’s lymphoma were not found (**Table 3**).

**Table 3:** Oral lesions in HIV-infected patients.

Lesion type	Cases	Percentage
Candidiasis albicans	51	47.23%
Salivary gland disease (xerostomia)	8	7.41%
Oral hairy leukoplakia	2	1.85%
Herpes simplex stomatitis	4	3.70%
AIDS-associated periodontitis	21	19.44%
Oral ulcers	22	20.37%

### Overall Characteristics of Roche 454 Sequencing in the Oral Flora in AIDS Patients After HAART

**Figure 1:**
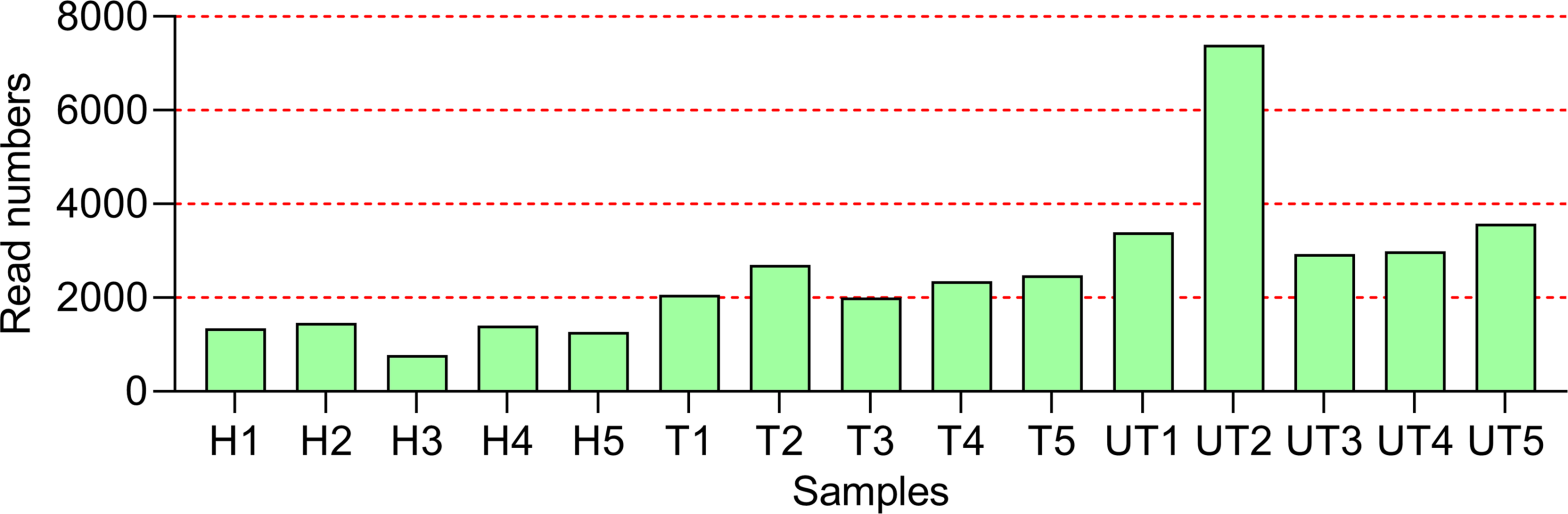
Overall characteristics of Roche 454 sequencing in the oral flora in HIV-infected/AIDS patients after HAART. The statistics of Read Number for each sample. H, Normal control; UT, HIV-infected/AIDS patients not processed with HAART; T, HIV-infected/AIDS patients processed with HAART.

Further, we adopted Roche 454 sequencing to investigate the impacts of HAART on the diversity of salivary microbial communities in 5 healthy controls, 5 HAART processed AIDS patients, and 5 HAART unprocessed AIDS patients. We obtained 150,342 valid 16s rDNA gene sequences. After the raw sequences were optimized and filtered, 81,182 high quality valid sequences were applied for data analysis. And the effective length of the sequences was around 241 bp after removing primer and Barcode (**Figure 1**).

### Diversity Analysis of Microecological Communities in the Saliva Samples of AIDS Patients After HAART

**Figure 2:**
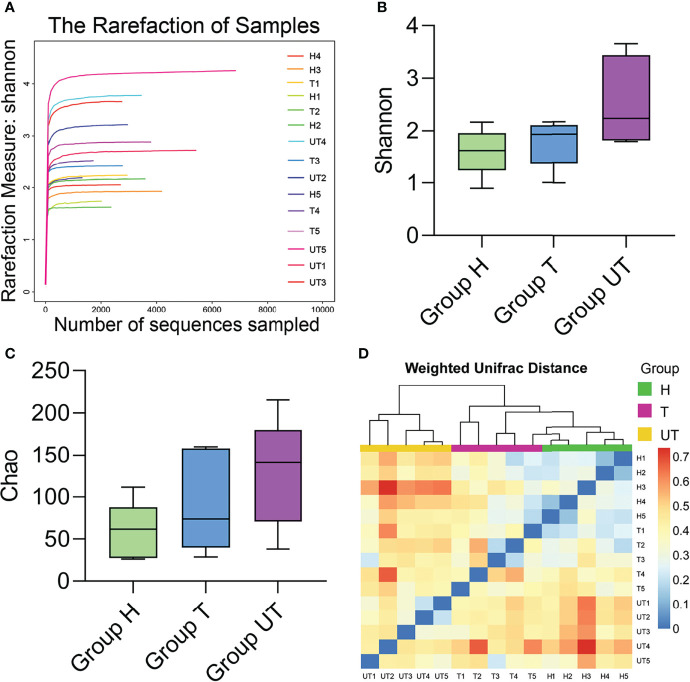
Diversity analysis of microecological communities in the saliva samples of HIV-infected/AIDS patients after HAART. **(A)** Rarefaction curve of Shannon index in the flora of saliva samples in different groups. **(B)** Comparison of Shannon index in HAART processed or unprocessed HIV-infected/AIDS patients. **(C)** Comparison of Chao1 index in HAART processed or unprocessed HIV-infected/AIDS patients. **(D)** Heat map of β-diversity the flora of saliva samples in different groups.

We first applied the rarefaction curve to evaluate the diversity of the original community. When the curve flattens out or reaches a plateau, the sequencing depth has largely covered all species in the sample. Our data displayed that the sequencing level has reached a good depth at the 0.03 cut off level. With the increase of sequencing volume, the shannon index curve gradually became flat. Therefore, our sequencing volume can basically reflect the diversity of oral flora in each group (**Figure 2A**). The α-diversity reflects the species diversity of individual samples and mainly includes: Shannon index and Chao1 index. The Shannon index was adopted to estimate the level of community diversity, and the higher the shannon value, the higher the community diversity. The chao1 index can assess the number of OUTs contained in the community by calculating the colony abundance, which responds to the total number of species. Our results denoted that the *P* values corresponding to shannon and Chao indices were less than 0.05, indicating the significant differences in community diversity from each group (**Figures 2B, C**). Besides, β-diversity can be applied to compare the differences between multiple groups of samples. And we compared the salivary microbial community diversity of AIDS patients using the UniFrac weighting algorithm to plot Heat map. Vertical clustering signifies the similarity of all species in samples, and horizontal clustering signifies the similarity in abundance of the species in samples. Based on the UniFrac clustering analysis of β-diversity of salivary microecological communities, we found that samples from healthy controls, samples from before treatment of HAART-processed AIDS patients, and samples from after treatment of HAART-processed AIDS patients were clustered together respectively (**Figure 2D**). Therefore, the microecological communities in the saliva of the three groups of patients had their own characteristics.

### Species Composition and Distribution of Flora in Saliva Samples From AIDS Patients After HAART at the Genus Level

**Figure 3:**
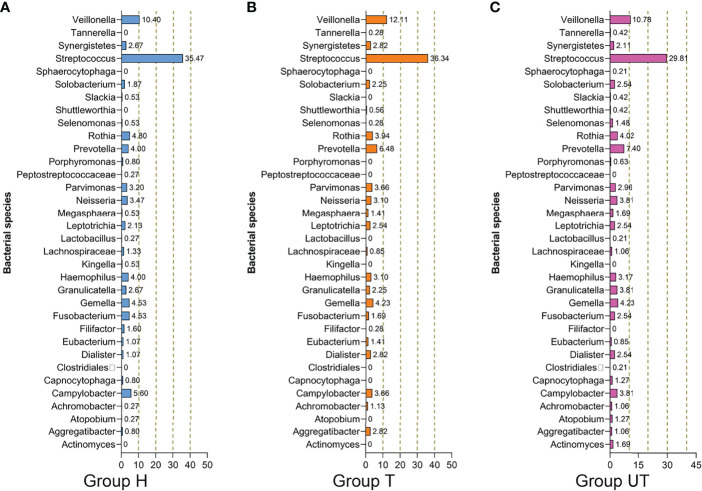
Species composition and distribution of flora in saliva samples from HIV-infected/AIDS patients after HAART at the genus level. Proportional distribution of major bacterial genus in the healthy control, HAART unprocessed, and HAART groups. **(A)** Bacterial species in Group H. **(B)** Bacterial species in Group T. **(C)** Bacterial species in Group UT. Group H, Healthy control; Group T, After treatment of HAART-processed AIDS patients; Group UT, Before treatment of HAART-processed AIDS patients.

Additionally, based on the sequencing alignment results, we discovered that there are 10 phyla of bacteria at the level of taxonomic “phylum” of biological species. Among them, *Firmicutes*, *Bacteroidetes*, *Proteobacteria*, *Fusobacteria*, and *Actinobacteria* accounted for more than 90% of the total bacterial phyla. And the differences between the three groups of samples were not statistically significant at this level of classification (**Table 4**). Moreover, at the “genus” level, we detected a total of 105 genera. And three of these genera (*Streptococcus, Synergistetes*, and *Veillonella*), were detectable in all three sets of saliva samples. The distribution of *Solobacterium*, *Gemella*, *Rothia, Neisseria*, *Leptotrichia*, *Parvimonas, and Haemophilus* was not significantly different in the three groups of samples. *Kingella*, *Slackia*, *Capnocytophaga*, *Peptostreptococcaceae*, *Atopobium*, *Porphyromonas*, and *Lactobacillus* were only present in the healthy control group. And some genera, such as *Fusobacterium*, *Selenomonas*, *Campylobacter*, *Capnocytophaga*, *Prevotella*, and *Granulicatella* were significantly increased in the samples from after treatment of HAART-processed AIDS patients compared with samples from before treatment of HAART-processed AIDS patients (**Figure 3**).

**Table 4:** Relative abundance of microbial diversity in saliva samples at the phylum level for different subgroups.

Phylum	Relative Distribution %		
	Group H	Group T	Group UT
*Actinobacteria*	8.98	9.01	8.09
*Bacteroidetes*	12.81	12.82	11.89
*Deinococcus-Thermus*	0	0.21	0
*Fusobacteria*	6.68	4.24	5.05
*Proteobacteria*	10.12	11.12	10.97
*Spirochaetes*	2.86	1.73	3.87
*Tenericutes*	0	0.12	0.21
*Firmicutes*	58.44	58.81	58.91
*Other*	0.11	0.96	1.01
*SR1*	0	0.98	0

Group H, Healthy control; Group T, After treatment of HAART-processed AIDS patients; Group UT, Before treatment of HAART-processed AIDS patients; HAART, Highly active antiretroviral therapy.

### Distribution of Saliva Sample Flora Species in AIDS Patients After HAART

**Figure 4:**
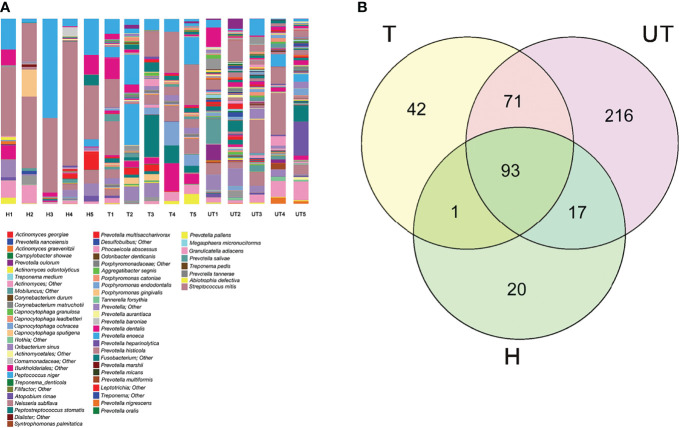
Distribution of saliva sample flora species in HIV-infected/AIDS patients after HAART. **(A)** The proportion of different species was displayed using the species profiling in each sample. **(B)** Venn diagram showed the overlap and differences in the composition of salivary microecological communities of different groups.

Furthermore, we also analyzed the distribution of flora species at the species level in three groups of saliva samples. As exhibited in **Figure 4A**, at the level of “species”, a total of 91 species were annotated in the saliva of healthy controls, 185 species in saliva samples from before treatment of HAART-processed AIDS patients, and 136 species in saliva samples of after treatment of HAART-processed AIDS patients. In the Venn diagram, we more intuitively understand the common presence of 93 OTUs in the three groups with significant diversity variation; there were 216 unique OTUs in the HAART unprocessed group, and these species might be associated with opportunistic infections (**Figure 4B**).

### Distribution of Different Genera of Bacteria in Saliva Samples of AIDS Patients After HAART Treatment

Based on the sequencing alignment results, RT-qPCR was performed to confirm the distribution of different genera of bacteria in saliva samples of AIDS patients after HAART treatment. As shown in **Table 5**, the positive samples of *Kingella*, *Slackia*, *Capnocytophaga*, *Peptostreptococcaceae*, *Atopobium*, *Porphyromonas*, and *Lactobacillus* were significantly lower in AIDS patients compared with healthy control group, meanwhile which were increased after HAART treatment. Furthermore, the contributions of *Fusobacterium*, *Campylobacter*, *Prevotella*, *Capnocytophaga*, *Selenomonas*, and *Granulicatella* in AIDS samples were significantly decreased in AIDS patients compared with healthy control group, meanwhile which were increased after HAART treatment (**Table 6**). The RT-qPCR results were consistent with the Roche 454 sequencing alignment results.

**Table 5:** Different genera of bacteria in saliva samples of AIDS patients after HAART treatment.

Genura	Positive		
	Group H (n = 78)	Group UT (n = 64)	Group T (n = 62)
*Capnocytophaga*	68 (87.18%)	8 (12.50%)^**^	51 (82.26%)^##^
*Slackia*	67 (85.90%)	12 (18.75%)^**^	48 (77.41%)^##^
*Porphyromonas*	72 (92.31%)	7 (10.94%)^**^	43 (69.35%)^##^
*Kingella*	74 (94.87%)	15 (23.44%)^**^	49 (79.03%)^##^
*Peptostreptococcaceae*	71 (91.03%)	14 (21.88%)^**^	51 (82.26%)^##^
*Lactobacillus*	69 (88.46%)	13 (20.31%)^**^	48 (77.42%)^##^
*Atopobium*	73 (93.59%)	10 (15.63%)^**^	42 (67.74%)^##^

Group H, Healthy control; Group T, After treatment of HAART-processed AIDS patients; Group UT, Before treatment of HAART-processed AIDS patients; HAART, Highly active antiretroviral therapy. **P < 0.01 vs Group H; ^##^P < 0.01 vs Group T.

**Table 6:** Different genera of bacteria in different saliva samples.

Genura	Contribution (%)		
	Group H (n = 78)	Group UT (n = 64)	Group T (n = 62)
*Fusobacterium*	6.27 ± 0.74	1.49 ± 0.41^**^	2.83 ± 0.47^##^
*Campylobacter*	8.62 ± 0.58	3.27 ± 0.65^**^	5.34 ± 0.46^##^
*Prevotella*	4.26 ± 0.41	2.32 ± 0.34^**^	3.86 ± 0.37^##^
*Capnocytophaga*	1.24 ± 0.21	0.24 ± 0.11^**^	0.12 ± 0.17^##^
*Selenomonas*	0.76 ± 0.13	0.26 ± 0.08^**^	0.57 ± 0.12^##^
*Granulicatella*	3.58 ± 0.52	1.38 ± 0.47^**^	2.86 ± 0.33^##^

Group H, Healthy control; Group T, After treatment of HAART-processed AIDS patients; Group UT, Before treatment of HAART-processed AIDS patients; HAART, Highly active antiretroviral therapy. **P < 0.01 vs Group H; ^##^P < 0.01 vs Group T.

## Discussion

Different opportunistic infections can invade different tissues and organs of the body (Duff, 2019). And the oral cavity is a common infection site for AIDS opportunistic infections (Peacock et al., 2017). AIDS-related oral lesions are considered the key indications for the detection and diagnosis of HIV infection (Keijser et al., 2008; Nasidze et al., 2009; Lazarevic et al., 2010; Li et al., 2010; Ling et al., 2010). Periodontal disease is a group of periodontal support tissue infections caused by oral bacteria and is the most frequent microbial infectious disease (Carrizales-Sepúlveda et al., 2018). Studies demonstrated a correlation between the occurrence of periodontal lesions and HIV infection, reflecting a close relationship between oral and systemic status (Patton et al., 2000; Paster et al., 2002; Vernon et al., 2009; Vernon et al., 2011).

In our study, we performed statistics on the relationship between periodontal and dental health indicators and demographic variables of HIV-infected/AIDS patients. The data displayed that age, sex, and marital status were associated with several periodontal health indicators. Although study has concluded that there is no statistical relationship between marital status and periodontal health status (Persson et al., 2004) a series of events such as family relationship breakups and financial pressures leading to increased mental stress in HIV-infected individuals may have had an impact on periodontal disease (Leserman et al., 1999; Ketchen et al., 2009; Dalmida et al., 2013; Warren et al., 2014). Meanwhile, socioeconomic variables such as income, education level, and residence were correlated with PD and CAL. And these results are consistent with the published literatures (Ababneh et al., 2012; Soares et al., 2013; Soares et al., 2014). Besides, our data revealed the percentage of BOP positive loci was lower in the group with lower BMI, probably because of the poorer systemic nutritional status of this group of patients. Researches confirmed that smoking can greatly increase the risk of periodontal destruction (Johnson, 2017); alcohol consumption is also a risk factor for developing periodontitis (Ryder et al., 2019). Our data further reveal an association between smoking and alcohol consumption and periodontal health of HIV-infected patients. Also, HIV-infected patients with good oral hygiene behaviors had better periodontal health. Additionally, our data also manifested that age, marital status and BMI were associated with dental health. And mouth rinsing frequency was also\ associated with number of permanent sound teeth and DMFT. Our present study also found that the most common oral lesion among HIV-infected patients was oral candidiasis, as previously reported (Suryana et al., 2020). And oral ulcers are the second most frequent oral lesion after oral candidiasis. Other study also proved that ulcers in people with HIV/AIDS are larger, deeper and longer lasting than those in HIV recessive patients (Tappuni, 2020). Dry mouth was also found in some patients in this study, but no enlargement of the salivary glands was detected. Therefore, oral hygiene behaviors are key for maintaining periodontal health in HIV-infected patients.

Oral microbiomes, as a complex and abundant microbiota, play a vital role in maintaining a normal oral physiological environment (Gao et al., 2018). In the human body, oral microbiomes can interact with the body to affect oral health and some systemic diseases (Graves et al., 2019). HIV-infected/AIDS patients are in a state of chronic immune system compromise, which has a significant impact on the overall oral flora (Hegde et al., 2014). The widespread application of HAART has been reported to improve oral representations (Fidel et al., 2021). However, the effect of HAART on the oral flora of AIDS patients has not been clearly elucidated. In our study, we adopted Roche 454 sequencing to analyze the salivary microbial diversity of healthy controls, before and after treatment of HAART-processed AIDS patient. Our data suggested that salivary microbial diversity can be reduced in AIDS patients processed with HARRT. This may be related to the increase of *Candida* and other genera including *Dialister*, *Aggregatibacter*, *Atopobium*, *Actinomyces*, etc. Besides, we detected 105 genera in the three groups of samples. Of these, *Streptococcus* and *Veillonella* were the dominant groups. Although these two genera are oral commensals, they contain many vital species that may cause human infections (Moussa et al., 2021; Yuan et al., 2021). Interestingly, seven genera were present only in the healthy control group. Five genera notably increased in the AIDS group; Six genera including *Aggregatibacter* prominently increased in the HAART-processed group, and *Aggregatibacter* dramatically decreased in the HAART-processed group. At the “species” level, we identified 319 strains. 7% of the strains only appeared in healthy control group and 24% of the strains appeared only in the AIDS group. Some strains exhibited significant differences among the three groups of samples, covering *Atopobium spp*, *Actinomyces gerencseriae*, and *Aggregatibacter segnis*.

In this study, the 108 HIV-infected patients have poor periodontal and dental health, mostly with oral lesions, and the severity of which is related to age, gender, BMI, and degree of immunodeficiency. Salivary microflora species were increased in HIV-infected patients relative to healthy controls. And HARRT treatment can obviously reduce salivary microbial diversity in AIDS patients. Therefore, the maintenance of oral health and oral ecosystem balance in HIV-infected/AIDS patients are of great importance. And HAART has a key role in the balance of salivary microecology in AIDS patients. Early HAART has an irreplaceable role in improving the oral health status of AIDS patients, reducing oral lesions and prolonging the survival of AIDS. Although HAART treatment has a beneficial effect on salivary microecological balance in AIDS patients, its possible mechanism of action is not clear and needs further study. Besides, we will also further collect more information about HAART-treated patients for examining and comparing the periodontal or dental health of patients before and after treatment. The small sample size of patients included in the Roche 454 sequencing is also a potential limitation of the current study, and we will expand the sample size in future studies to focus on the diversity of salivary microbial communities in HIV-infected/AIDS patients.

## Data Availability Statement

The datasets presented in this study can be found in online repositories. The name of the repository and accession number can be found below: NCBI; PRJNA844037.

## Ethics Statement

The studies involving human participants were reviewed and approved by This study also received the approval of the ethics committee in West China Hospital of Stomatology, Sichuan University (WCHSIRB-D-2015-004). The patients/participants provided their written informed consent to participate in this study.

## Author Contributions

YL and YY conceived and designed the study and provided administrative support. PC, YZ and GD performed the experiments and analyzed data. PC, YZ and HW analyzed and interpreted the data. YL, GD and YY wrote the manuscript. All authors read and approved the final manuscript.

## Funding

This study was supported by Science and Technology Foundation of Chengdu, China (2021-YF05-00442-SN), Science and Technology Foundation of Sichuan Province, China (2022YFS0116).

## Conflict of Interest

The authors declare that the research was conducted in the absence of any commercial or financial relationships that could be construed as a potential conflict of interest.

## Publisher’s Note

All claims expressed in this article are solely those of the authors and do not necessarily represent those of their affiliated organizations, or those of the publisher, the editors and the reviewers. Any product that may be evaluated in this article, or claim that may be made by its manufacturer, is not guaranteed or endorsed by the publisher.
